# Multidrug resistance operon *emrAB* contributes for chromate and ampicillin co-resistance in a *Staphylococcus* strain isolated from refinery polluted river bank

**DOI:** 10.1186/s40064-016-3253-7

**Published:** 2016-09-22

**Authors:** He Zhang, Yantian Ma, Pu Liu, Xiangkai Li

**Affiliations:** 1 MOE Key Laboratory of Cell Activities and Stress Adaptations, School of Life Sciences, Lanzhou University, Tianshuinanlu #222, Lanzhou, 730000 Gansu People’s Republic of China; 2 School of Life Sciences, Nanchang University, Nanchang, 300031 Jiangxi People’s Republic of China

**Keywords:** *Staphylococcus aureus* LZ-01, Co-resistance, Chromate, Ampicillin, *emrAB* operon

## Abstract

*EmrAB* operon is known for multidrug resistance in bacteria and yet has not been reported related to heavy metal resistance or antibiotics/heavy metal co-resistance. Strain *Staphylococcus aureus* LZ-01 which was isolated from industrial wastewater discharging site can co-resist to 6 mM Cr(VI) and 0.75 mg/ml ampicillin. Transcriptome data showed that an *emrAB* operon was upregulated (1.29-folds for *emrA,* 2.14-folds for *emrB*) under 0.4 mM Cr(VI) treatment. Quantitative PCR results revealed that this operon was upregulated (1.60-folds for *emrA,* 2.34-folds for *emrB*) after 0.20 mg/ml ampicillin treatment. Mutant strain with *emrA* gene knockout resulted in a 0.83-folds decrease in chromate resistance, and a 0.80-folds decrease in ampicillin resistance; while *emrB* knockout strain resulted in a 0.33-folds decrease in chromate resistance, and a 0.60-folds decrease in ampicillin resistance. The complemented strains of both deletion mutants basically restored their resistant performance. The presence of 0.50 mM Cr(VI) induced an elevation in ampicillin resistance from 0.50 to 2.50 mg/ml in the strain LZ-01, similarly, its Cr(VI) resistance was also found to be elevated from 6 to 10 mM by 0.15 mg/ml ampicillin induction. The induction effect could be eliminated by deletion of *emrA* or *emrB*. Our results demonstrated that the chromosomal *emrAB* operon in *Staphylococcus aureus* LZ-01 was a new type of multidrug resistance system, which conferred both ampicillin and chromate resistance to host cells inhabiting polluted environments.

**Electronic supplementary material:**

The online version of this article (doi:10.1186/s40064-016-3253-7) contains supplementary material, which is available to authorized users.

## Background

Antibiotics were once the most powerful weapon against diverse diseases in human medical history since its discovery in early 20th century. But since the occurrence of vast antibiotic resistant pathogens in 1960s, a series of superbugs were found to have pan-drug resistance, like MRSA (Methicillin-resistant *Staphylococcus aureus*), NDM-1, vancomycin-resistant enterococci (VRE) and CA-MASA (Enright et al. 2002; Chambers and DeLeo 2009; Poirel et al. 2010; Datta et al. 2014). Abuse of antibiotics was used to be supposed as the major cause but the decreased usage of antibiotics subsequently does not necessarily prevent the spread of antibiotic resistance in clinical (Salyers and Amábile-Cuevas 1997). Meanwhile, many studies have found that antibiotic resistance positively correlated to toxic metals or other antimicrobials in environments (Seiler and Berendonk 2012). Metal-induced antibiotic resistance is supposed to be more prevalent than antibiotic itself, due to the fact that metals were more persistent than antibiotics in nature environment (Kolpin et al. 2002). The metal contamination is hard to eliminate due to the huge areas and scattered distributions, metal-induced antibiotic resistance are enriched either in amount or abundance under metal stress, and incurable diseases caused by resistant pathogens would be prevalent and become a disaster (Aminov 2010). Therefore, it is essential to fully understand the co-resistant mechanism of metals and antibiotics, and find ways to decelerate the spreading tendency of resistant genes.

Metal-induced co-resistant bacteria was frequently found in various metal contaminated environments, including shores polluted by hospital wastes and factory discharges (Matyar et al. 2010), livestock farms contaminated by animal feeding amendment (Hölzel et al. 2012), and other polluted water environments (Baker-Austin et al. 2006; Baquero et al. 2008). Compared with the above massive studies in community level, less research focused on the molecular bases of co-resistance or the mechanisms where co-resistance came from. Beside the gene linkage phenomenon of metal and antibiotic resistance on transferable elements which was found several decades ago, multidrug resistance (MDR) pump was another important mechanism explored afterwards (Foster 1983). Plasmids from *Salmonella abortus equi* contained resistances to ampicillin and several metals (Ghosh et al. 2000), the copper and macrolide resistance plasmid (*tcrB*-*ermB*) was found in *Enterococcus faecium* (Aarestrup et al. 2002; Hasman and Aarestrup 2002). The MexGHI-OpmD efflux pump in *Pseudomonas aeruginosa* could improve the bacterial resistance to vanadium and ticarcillin (Aendekerk et al. 2002), and DsbA-DsbB system in *Burkholderia cepacia* was involved in metal-efflux and multi-drug resistance (Hayashi et al. 2000). *EmrAB* operon was another well-studied MDR pump in *Escherichia coli* (Lomovskaya and Lewis 1992), and was also reported in *Aquifex aeolicus*, *Rhodobacter capsulatus*, *Salmonella typhimurium* etc. (Xiong et al. 2000). *EmrAB* could extrude structurally unrelated compounds such as oxidative salicylic acid, 2,4-dinitrophenol (DNP) and carbonyl cyanide m-chlorophenylhydrazone (CCCP) out of cell membrane (Hinchliffe et al. 2014), but no metals or β-lactams was found as inducer that might enhance the expression of *emrAB* so far. Moreover, the most studied strains for *emrAB* belonged to Gram-negative phenotype, and we have little examples for Gram-positive bacteria.

*Staphylococcus aureus* is a prevalent gram-positive bacterium in clinical, it is best known for its ability to cause a range of illnesses and resist to vast antibiotics (Ito et al. 2003). The *S. aureus* strain LZ-01 was isolated from industrial contaminated river bank soil of the Yellow River in Lanzhou city (zhang et al. 2013). It has the ability to grow in chromate amendment medium aerobically, and further study proved its antibiotic resistant potential. So in this study we aimed to find the connection between metal resistance and antibiotic resistance of strain LZ-01. The RNA sequencing, qPCR and gene knockout methods were employed to find crucial genes that conferring metal and antibiotic co-resistance. Our results indicated that an operon *emrAB* consisted of two genes- SAV2352 and SAV2353, contributed to the co-resistance of antibiotic and metal in *S. aureus* LZ-01. This study expands our knowledge of versatile functions of *emrAB* operon, and helps to understand the mechanism of metal and antibiotic co-resistance at a genetic level.

## Methods

### Bacterial strains and cultural conditions

The *Staphylococcus aureus* LZ-01 was isolated from the contaminated river bank soil of Lanzhou Reach of the Yellow River, the sampling site was polluted by waste water from a oil refinery (Zhang et al. 2013). Cultural medium used for the growth of strain LZ-01 was TSB medium. TSB medium contained: 1.7 % (W/V) tryptone, 0.3 % (W/V) soytone, 0.25 % (W/V) glucose, 0.5 % (W/V) NaCl and 0.25 % (W/V) K_2_HPO_4_. The strain was incubated at 37 °C and shaked 180 rpm. Bacterial growth was monitored by OD_600_.

### Determination of metal and antibiotic resistance

The concentration gradients of Cr(VI), As(V), Hg(II), Pb(II), Mn(II) and Cu(II) were prepared in TSB medium with stock solutions. The stock solutions of different metals were prepared in deionized water and autoclaved at 120 °C for 20 min or filtration sterilized with 0.15 µm membrane. The concentration was 0.5 M for K_2_Cr_2_O_7_, 0.5 M for H_3_AsO_4_·12H_2_O, 0.1 M for HgCl_2_, 0.5 M for Pb(NO_3_)_2_, 0.5 M for MnCl_2_·4H_2_O and 1 M for CuSO_4_·5H_2_O.

The TSB medium contained different kinds of antibiotics was prepared for antibiotic resistance test, the antibiotics and their concentrations used in this test were as follows: ampicillin (0–5 mg/ml), chloramphenicol (0–1 mg/ml), vancomycin (0–1 mg/ml), kanamycin (0–1 mg/ml), erythromycin (0–500 μg/ml), gentamycin (0–500 μg/ml) and tetracycline (0–500 μg/ml). The growth status under different antibiotics was measured by both visual inspection and optical density OD_600_.

All strains were incubated at 37 °C, 180 rpm for 12–16 h to obtain log phase culture. All the treatments were conducted using above mediums, and cultured in a shaker with 180 rpm and 37 °C, this procedure lasted up to 36 h. The final result was confirmed by three times individual tests.

### The induction and cross-tolerance tests of strain LZ-01 and mutants

The TSB medium was prepared for the chromate induced test. Briefly, strains were cultured in TSB medium with 0.5 mM hexavalent chromate and incubated at 37 °C, 180 rpm for 8–12 h to obtain an optical density OD_600_ at 0.4. A volume of 100 μl of above chromate induced cultures was pipeted into a series of tubes with 5 ml TSB medium and a gradient of ampicillin concentrations (0–5 mg/ml). The growth status under different ampicillin concentrations was measured by visual inspection.

The procedure for manganese induced test was just as chromate induced test described above. The induced level of manganese was 0.5 mM. For antibiotic induced test, the procedure was similar to metal induction, except for the first step with different antibiotic induced levels for different strains. The induced level of ampicillin for strain LZ-01 was 0.15 mg/ml, for mutants was 0.05 mg/ml. The induced level of chloramphenicol was 0.15 mg/ml.

The cross-tolerance tests were carried out using TSB medium supplemented with hexavalent chromium and ampicillin simultaneously. The concentration gradient for hexavalent chromium (mM) was 0.8, 1.6, 3.2, 4.0, 4.8, 6.0, 8.0 and 10.0; concentration gradient for ampicillin (mg/ml) was 0.1, 0.2, 0.4, 0.5, 0.6, 0.75, 1.0 and 1.25. The tested strain was cultured in TSB medium overnight, and the inoculation proportion was 1:50. The growth status under different ampicillin/chromate concentrations was measured by visual inspection and marked with ‘+’ (growth) or ‘−’ (no growth).

### RNA-seq analysis and qPCR assay

The transcriptional information of *S. aureus* strain LZ-01 after 0.4 mM Cr(VI) treatment was analyzed previously, and many genes were found to respond to the metal stress (Zhang et al. 2014). The differentially expressed gene between the mRNA of *S. aureus* LZ-01 with and without Cr(VI) was calculated as fold change referring to “The significance of digital gene expression profiles” (Audic and Claverie 1997). The most up-regulated and antibiotic resistance associated genes were selected out as candidates for the next qPCR test.

The *Staphylococcus aureus* LZ-01 was treated with 0.2 mg/ml ampicillin, and the total RNA was extracted from log phase cultures, then reverse transcribed to cDNA. Total RNA isolation was conducted as following: 0.5 ml overnight incubated cultures of *S. aureus* LZ-01 was diluted into 50 ml TSB medium and cultivated at 37 °C 180 rpm. When OD_600_ value was just over 0.2, the ampicillin was added to a final concentration of 0.2 mg/ml. The culture incubation was continued until OD_600_ value reached 0.4. 30 ml cultures were centrifuged at 10,000*g* for 3 min to harvest a cell pellet. Then total RNA was isolated from the pellet using the SV Total RNA Isolation System (Promega, USA) according to the manufacturer’s instructions. *Dnase*I was used to purify the extracted total RNA by digesting the residual DNA. A control sample without ampicillin treatment was collected for baseline control. All RNA preparations were quantified using a ND-1000 spectrophotometer (NanoDrop Technologies). The reverse transcription was conducted as follows: the reaction mixture was: 2 µl 5 × PrimerScript RT Master Mix, 1 µl RNA template and 7 µl RNase free dH_2_O (PrimerScript RT Master Mix Perfect Real Time, TaKaRa). The reaction condition was: 37 °C for 15 min and then 85 °C for 5 s. Primers were designed and synthesized to cover the selected candidate genes. All the primers were designed in Primer 5.0 software, and synthesized by Beijing Genomics Institution (BGI).

qPCR method was used to quantify the transcriptional level of these candidate genes under ampicillin stress, and cDNA from cultured strain LZ-01 without ampicillin was used as control. The detailed qPCR procedure was as follows: 12.5 µl SYBR *Primix Ex Taq*™ II, 1 µl PCR forward primer (10 µM), 1 µl PCR reverse primer (10 µM), 1 µl cDNA and 9.5 µl dH_2_O (SYBR *Primix Ex Taq*™II, TaKaRa). The quantification reaction was conducted under the following conditions: 30 s at 95 °C, followed by 95 °C 5 s, 58 °C 30 s, 40 cycles.

Five technical replicates of the qPCR reactions were performed for each sample. The housekeeping gene, 16S rRNA which appears to be constitutively expressed, was selected as the internal control for normalization. The primer efficiency of the amplifications for each gene was valued through a standard curve by serial dilution of cDNA. The efficiency was calculated according the formula E = 10^(−1/k)^ − 1, where k is the slope. The fold change of gene expression was calculated by 2^−ΔΔCt^ method and normalized relative to 16S rRNA (Livak and Schmittgen 2001).

### Gene knockout and complementation

The mutation strains of *S. aureus* LZ-01 were constructed using a strategy that used the temperature-sensitive allelic replacement shuttle vector pMAD (Arnaud et al. 2004). The upstream and downstream sequences that flanking the *emrA* and *emrB* fragments were amplified from strain LZ-01 by PCR. The primers used were *emrA*-up-F and *emrA*-up-R for the *emrA* upstream fragment and *emrA*-down-F and *emrA*-down-R for the downstream fragment. Primers used were *emrB*-up-F and *emrB*-up-R for the *emrB* upstream fragment and *emrB*-down-F and *emrB*-down-R for the downstream fragment. The neomycin phosphotransferase II gene (*npt* II) fragment coding kanamycin resistance was PCR amplified from plasmid pET-30a by using the primers *npt* II-F and *npt* II-R (Table 1). Three fragments including amplified upstream arm, *npt* II and downstream arm were linked together using Double-joint PCR method (Yu et al. 2004).

**Table 1:** The primers, strains and plasmids used in this study

Primers, strains and plasmids	Sequence (5′–3′) or description	Source or reference
Primers for qPCR (Supplied in Additional file 1: Table S1)		
Primers for gene knockout (Extended sequences for overlap are marked with underline)		
*EmrB*-up-F	TTAACTAGACAGATCTATTTCTATTTTGGCTTGTCGTT	This study
*EmrB*-up-R	AAAATCCCTTAACGTGAGTTCCTTTTGTTGGCGTTTGC	This study
*EmrB*-down-F	CCGAAAAGTGCCACCTGAAATATGGTGGTCAAGAAGGCG	This study
*EmrB*-down-R	GGGCGATATCGGATCCCTGGATACAGCATGTGGAAAC	This study
*EmrA*-up-F	TCGATGCATGCCATGGAAATGTCAGCAACTTCTTCAGG	This study
*EmrA*-up-R	AAAATCCCTTAACGTGAGTTAATAAAAGCCAGCAATCCCAAT	This study
*EmrA*-down-F	CCGAAAAGTGCCACCTGAAATCCTGGAATGAACGCTGAAG	This study
*EmrA*-down-R	GGGCGATATCGGATCCAAATAGATACGCCGTAATTGGTA	This study
*npt* II-F	AACTCACGTTAAGGGATTTTGG	This study
*npt* II-R	TTTCAGGTGGCACTTTTCG	This study
Strains used in this study		
*S. aureus* LZ-01	*S. aureus* LZ-01 is the isolated objective strain	Zhang et al. (2013)
*S. aureus* ATCC 25923	*S. aureus* ATCC 25923 is a purchased standard strain	Purchased from CCTCC
*S. aureus* RN4220	Initial recipient for modification of reconstructed plasmids	Augustin and Götz (1990)
*E.coli.* Top 10	*E.coli.* Top10 is used as a host for vector replication	University of Chicago
Δ*emrB*	Δ*EmrB* is *S. aureus* LZ-01 mutant strain with deletion of *emrB*	This study
Δ*emrA*	Δ*EmrA* is *S. aureus* LZ-01 mutant strain with deletion of *emrA*	This study
CB	*S. aureus* CB is the complemented strain for Δ*emrB*	This study
CA	*S. aureus* CA is the complemented strain for Δ*emrA*	This study
Plasmids		
pMAD	pMAD is a shuttle vector used for gene knockout of *S. aureus*	Arnaud et al. (2004)
pET-30a	pET-30a used for clone of *npt* II gene	Purchased

The constructed fragments of *emrA* upstream-*npt* II- *emrA* downstream and *emrB* upstream-*npt* II- *emrB* downstream were ligated into pMAD and transformed into *E. coli* Top 10. The plasmid pMAD was digested with the enzyme *Nco*I and *Bam*HI before ligation. The ligation was performed by using an In-Fusion system (In-Fusion^®^ HD Cloning Kit, TaKaRa) following the manufacturer’s instructions. Plasmid DNA obtained from *E.coli* Top 10 was then transformed into the intermediate *S. aureus* host strain RN4220 by electroporation (Augustin and Götz 1990). Plasmid DNA harvested from strain RN4220 was then transformed into *S. aureus* LZ-01, and the mutant screening was conducted (Jo et al. 2011). Deletion of the gene from chromosome was confirmed by DNA sequencing of a PCR fragment from transformants that was obtained using the primers corresponding to the upstream and downstream sequences of the deleted gene. To verify restoration of the resistant phenotype of *S. aureus* LZ-01, we complemented the mutant strains. The PCR fragment containing the whole operon expressed from its own promoter was cloned into plasmid pMAD as described previously. The plasmid was introduced into mutants to create strain LZ-01.

## Results

### *S. aureus* LZ-01 resists to several heavy metals and antibiotics

Given that *S. aureus* LZ-01 was isolated from the contaminated river bank soil near oil definery, we supposed that the strain may possess different response to heavy metals. To test this hypothesis, we compared the resitant level of LZ-01 and ATCC standard strain (*S. aureus* ATCC 25923) to several heavy metals, including Cr(VI), As(V), Hg(II), Pb(II), Mn(II) and Cu(II). The resistance of strain LZ-01 to As(V), Hg(II), Pb(II) and Cu(II) was comparable to that of the ATCC 25923. However, The strain LZ-01 had 2- and threefold increases in resistance to Cr(VI) and Mn(II) when compared to the strain ATCC 25923 (Table 2).

**Table 2:** The MIC levels of antibiotics and heavy metals in different strains

Strains	Antibiotics (mg/ml)^a^		Metal concentrations (mM)					
	Amp	Chlo	Cr(VI)	As(V)	Hg(II)	Pb(II)	Mn(II)	Cu(II)
LZ-01	0.51	0.25	6	4	0.005	1	6	1
ATCC25923	0.06	0.13	3	4	0.005	0.05	2	1
Δ*emrB*	0.15	0.25	4	4	0.005	0.5	4	1
Δ*emrA*	0.10	0.30	1	3	0.005	0.5	4	1
CB	0.51	0.26	5	4	0.005	0.5	6	1
CA	o.51	0.26	5	4	0.005	0.5	5	1

^a^All the data here represent consistent result of 3–5 individual tries of gradient dilution series

**Fig. 1:**
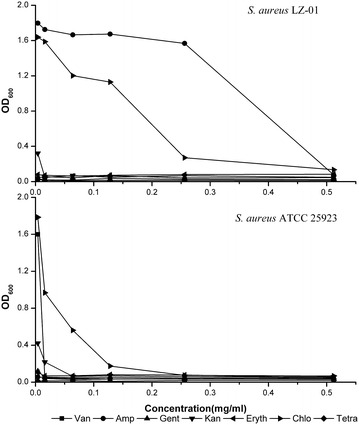
The comparison of antibiotic resistant levels between strain *S. aureus* LZ-01 (the *upper part*) and type strain *S. aureus* ATCC 25923 (the *lower part*)

The antibiotic resistant level of strain LZ-01 was also evaluated in comparison to the typical strain *S. aureus* ATCC 25923. Strain LZ-01 had high ampicillin and chloramphenicol resistance (0.51 and 0.25 mg/ml respectively), but strain ATCC 25923 only have partial chloramphenicol resistance (0.13 mg/ml) and little vancomycin resistance (0.02 mg/ml). Both strains were sensitive to other antibiotics including kanamycin, erythromycin, gentamycin and tetracycline, and the MICs for these antibiotics were less than 0.004 mg/ml (Fig. 1). These results showed that *S. aureus* LZ-01 was mostly resistant to ampicillin, chromate and manganese, and subsequently resist to chloramphenicol and Pb(II).

### The *emrAB* operon was involved in co-resistance to chromium and ampicillin

Based on the antibiotic and metal resistant performance showed above, we were attracted to explore the handlers behind this performance in *S. aureus* LZ-01. The genes involved in drug/multidrug/antibiotic transport/resistance and efflux pump were selected from RNA sequencing database of strain LZ-01 which was pre-treated with 0.4 mM chromium. These genes which up-regulated under chromate pressure were quantified by qPCR method in the LZ-01 cultures before and after ampicillin treatment (0.2 mg/ml). Concerning the conventional ampicillin resistance, the genes related to beta-lactamase and penicillin-binding proteins were also selected for qPCR tests. A total of 40 genes were finally selected and described in Table 3.

**Table 3:** Genes involved in co-resistance of Cr(VI) and ampicillin were tested by RNA-seq and qPCR methods in strain LZ-01

Genes	Gene annotation	RNA-seq (0.4 mM Cr(VI)) Fold change	qPCR (0.2 mg ml^−1^ Amp) Fold change
Multidrug transporter and resistance associated genes			
SAV0198	ABC-type antimicrobial peptide transport system	6.21	0.61
SAV0199	ABC-type antimicrobial peptide transport system	10.14	0.72
SAV0203	ABC-type multidrug transport system	7.92	0.86
SAV0274	Drug transporter	5.78	0.57
SAV0275	Penicillin amidase V	2.96	0.72
SAV0276	Peptidoglycan hydrolase	1.58	0.47
SAV0277	ABC-type multidrug transport system	1.14	0.79
SAV0351	ABC-type multidrug transport system	1.07	1.18
SAV0661	ABC-type antimicrobial peptide transport system	1.86	1.19
SAV1035	ABC-type antimicrobial peptide transport system	8.23	0.30
SAV1318	ABC-type multidrug transport system	5.10	0.97
SAV1837	ABC-type multidrug transport system	1.01	0.99
SAV1866	ABC-type multidrug transport system	1.23	0.71
SAV2166	Drug resistance MFS transporter	1.47	0.58
SAV2168	Putative multidrug transporter	4.26	1.05
SAV2169	Multidrug ABC transporter	3.58	1.01
SAV2261	Cation/multidrug efflux pump	1.25	1.13
SAV2262	Uncharacterized protein involved in methicillin resistance	1.01	1.12
SAV2265	Mar R family transcriptional regulator	10.64	0.67
SAV2266	MFS family transporter ybf D	6.44	0.63
SAV2352(*emrB*)	Drug resistance MFS transporter, drug:H^+^ antiporter-2 family	2.14	2.34^a^
SAV2353(*emrA*)	Multidrug resistance efflux pump	1.29	1.60^a^
SAV2355	Drug resistance transporter, Bcr/Cfl A subfamily	3.60	0.86
SAV2419	Toxin production and resistance	1.28	0.94
SAV2420	Toxin production and resistance	1.22	0.94
SAV2421	Toxin production and resistance	1.51	0.59
SAV2428	ABC-type multidrug transport system	15.80	0.51
SAV2462	Antibiotic resistance protein	2.13	0.79
SAV2552	Predicted drug exports of the RND family	4.55	0.66
SAV2623	ABC-type antimicrobial peptide transport system	1.68	0.96
SAV2701	ABC-type antimicrobial peptide transport system	13.05	0.73
SAV2702	Similar to ABC transporter, vraE protein	10.66	0.63
Ampicillin-resistant genes			
SAV1275	Metallo-beta-lactamase superfamily	0.98	0.80
SAV1504	Metallo-beta-lactamase	1.02	0.51
SAV1545	Metallo-beta-lactamase superfamily protein	0.74	1.02
SAV2441	Similar to beta-lactamase	1.42	0.44
*pbpA*	Penicillin-binding protein 1	1.54	3.88
*pbp2*	Penicillin-binding protein 1A	2.10	2.02
*pbp3*	Penicillin-binding protein 3	1.05	2.71
*pbp4*	d-alanyl-d-alanine carboxypeptidase (penicillin-binding protein)	0.77	3.32

^a^Stands for the selected genes for next study

Although the RNA-seq data proved the expression increase of selected genes (1.01-fold to 15.80-fold change), most of the selected genes failed to increase their expression in qPCR test under ampicillin stress. Only 9 genes were observed that their expression increased with the fold change over 1.00, and among which *SAV2352* and *SAV2353* were highlighted due to their most up-regulated expressions. Based on the homology blast results, *SAV2352* and *SAV2353* were named *emrB* and *emrA* in this study (Additional file 1: Figure S1). The gene *emrB* increased its expression quantity 2.14 times under hexavalent chromium stress, and increased its expression quantity 2.34 times under ampicillin stress, thus *emrB* was supposed as the key gene functioned in the co-resistance of metals and antibiotics in strain LZ-01. Gene *emrA* was aligned just behind *emrB*, and both trans-arranged in an operon in the genome of strain LZ-01. Gene *emrA* increased its expression quantity 1.29 times under chromium stress, and increased its expression quantity 1.60 times under ampicillin stress (Table 3). The genes associated with ampicillin-resistance were also focused, among which *pbpA*, *pbp2*, *pbp3* and *pbp4* had positive response to ampicillin stress.

### Deletion of *emrA* or *emrB* decreases both resistance to chromate and ampicillin

**Fig. 2:**
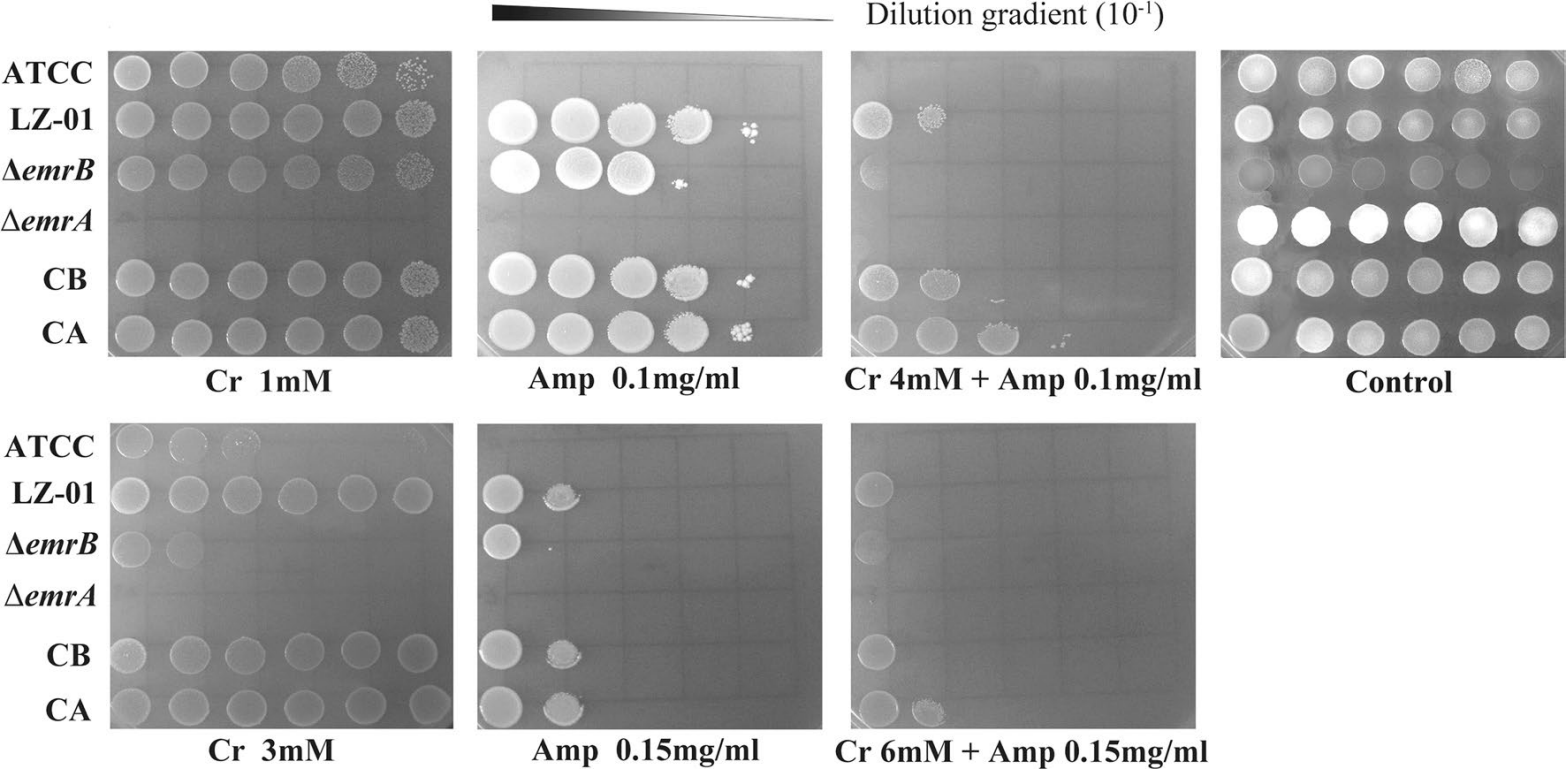
The growth status of different strains on the TSA medium with Cr(VI) and/or ampicillin added. The control means no Cr(VI) or ampicillin was added. ATCC stands for strain *S. aureus* ATCC25923, LZ-01 stands for strain *S. aureus* LZ-01, CA and CB are complemented strains for deletion mutant strain Δ*emrA* and Δ*emrB*. Cultures of different strains were diluted to tenfold gradients. 3 µl of each gradient culture was inoculated on to plate in a line, and different strains were inoculated in a column. All the plates were incubated in 37 °C for 12–18 h

**Fig. 3:**
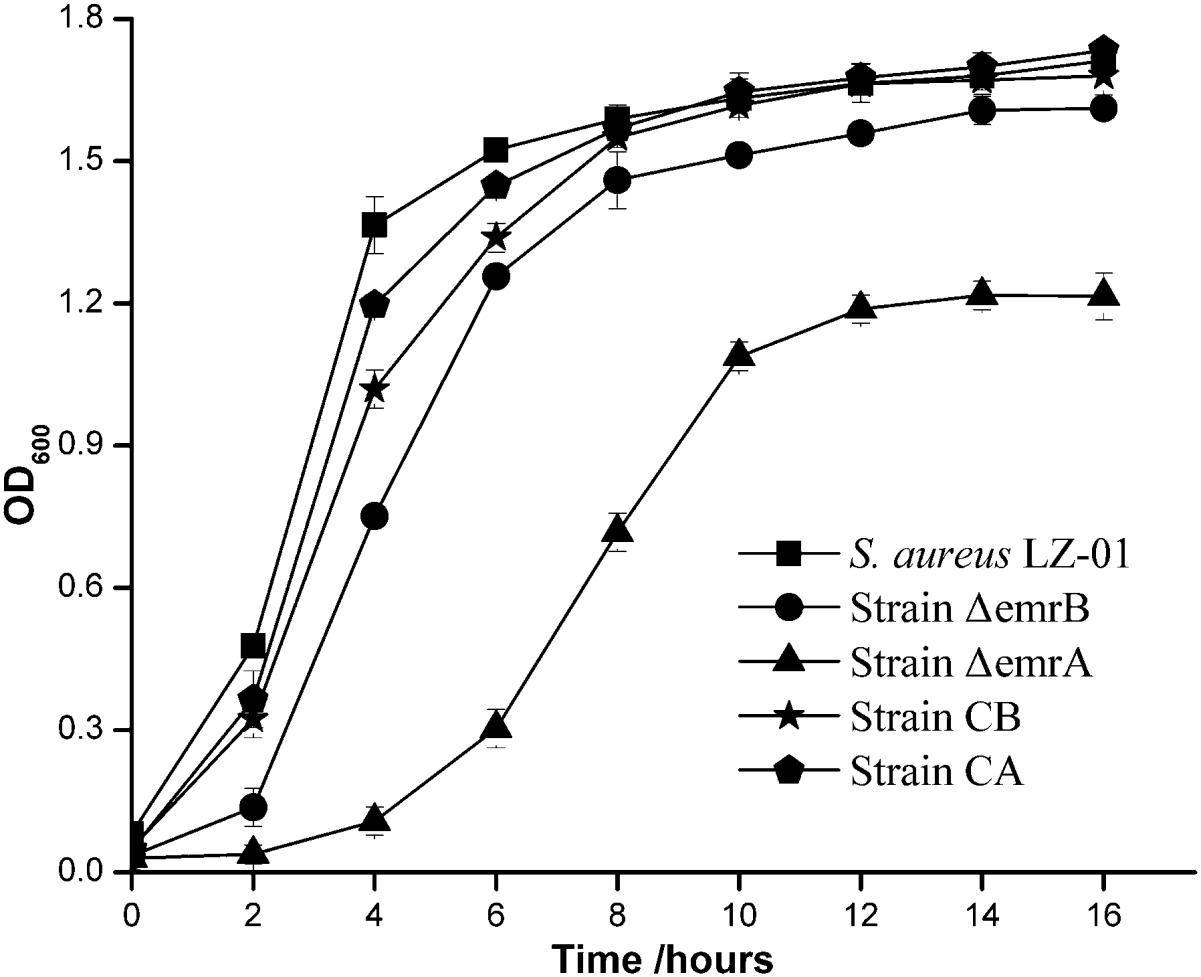
The growth curves of strain *S. aureus* LZ-01 and mutants

Both RNA-seq and qPCR tests proved that the *emrAB* operon was responsible for co-resistance of ampicillin and chromate in *S. aureus* LZ-01. Thus gene knockout test of *emrA/B* was conducted to verify their roles in co-resistance. The deletion of *emrA/B* from strain LZ-01 changed its original morphology on agar plate (Fig. 2), and the growth curve also showed growth defects of deletion mutants (Fig. 3). The cell doubling time of mutants extended and cell density declined, especially for *emrA* deletion mutant. The deletion of *emrB* increased susceptibility to ampicillin more obviously than to chromate, while strain Δ*emrA* increased susceptibility to both chromate and ampicillin. The growth status of mutant Δ*emrA* and Δ*emrB* were different on plates with chromate and ampicillin pressures (Fig. 2).

Compared to strain LZ-01, the metal tolerance level of Δ*emrB* decreased, the MICs for Cr(VI) and Mn(II) both decreased from 6 to 4 mM, the MIC for Pb(II) decreased from 1 to 0.50 mM (Table 2). The ampicillin resistance level of Δ*emrB* also decreased from 0.5 to 0.15 mg/ml. The deletion of *emrA* caused more severe drop in metal tolerance compared with Δ*emrB*, the MIC for Cr(VI) decreased from 6 to 1 mM, the MIC for As(V) decreased from 4 to 3 mM, the MIC for Pb(II) decreased from 1 to 0.50 mM, and the MIC for Mn(II) decreased from 6 mM to 4 mM. The ampicillin resistance level of Δ*emrA* decreased from 0.50 to 0.10 mg/ml (Table 2). The complemented strains CB and CA have recovered most resistance level to ampicillin(1/1) and chromate(5/6) compared with the wild strain LZ-01. The resistant level of chloramphenicol, Mn(II) and Pb(II) were not significantly changed among the wild strain LZ-01, mutant strain Δ*emrA* and Δ*emrB*, and complemented strain CB and CA. These results indicated that *emrAB* are primarily responsible for the resistances of both Cr(VI) and ampicillin in *Staphylococcus aureus* LZ-01.

### The chromate and ampicillin co-resistance of *Staphylococcus aureus* LZ-01 could be positively induced by chromate or/and ampicillin

**Fig. 4:**
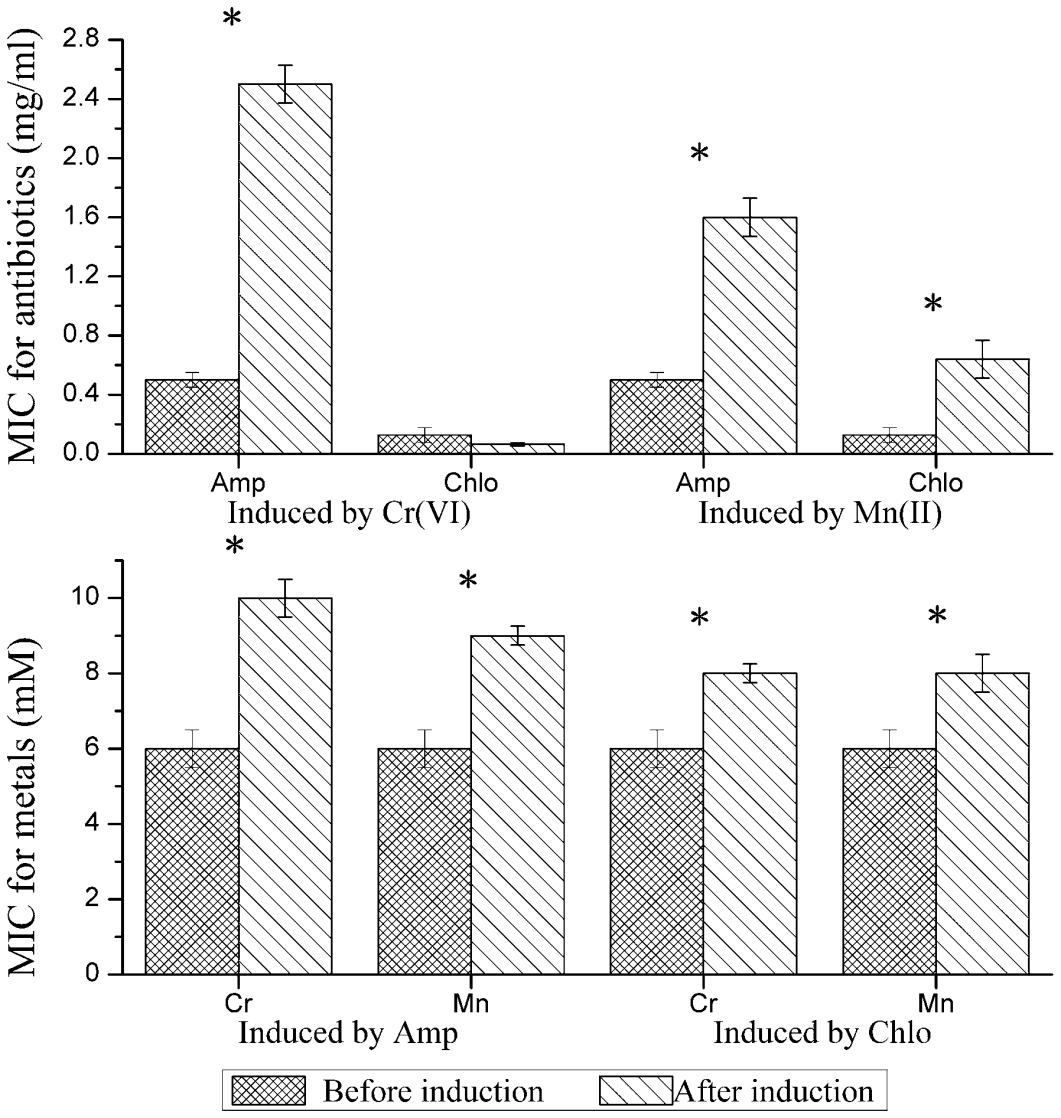
Induction effects on MICs for antibiotics and metals on strain *S. aureus* LZ-01. The induced level was 0.15 mg/ml for Amp, 0.05 mg/ml for Chlo, 0.50 mM for Cr(VI) and Mn(II). *Asterisk* means significant difference between two groups (*T* tests, p < 0.05)

Since the strain LZ-01 has exhibited higher resistance level to metal chromium and antibiotic ampicillin, we wondered that whether these two resistances could be induced by each other in strain LZ-01. When the strain LZ-01 was pre-treated with 0.15 mg/ml ampicillin, its chromate resistance level improved 2/3 times, from 6 to 10 mM; the manganese resistance level also increased from 6 mM to 9 mM. The pre-treatment of strain LZ-01 with 0.05 mg/ml chloramphenicol also showed improved metal resistance, the resistance of both chromium and manganese increased to 8 mM. When the strain LZ-01 was pre-treated with 0.50 mM hexavalent chromium, its ampicillin resistance level improved almost 5 times, from 0.50 to 2.50 mg/ml; but the chloramphenicol resistance level decreased from 0.13 to 0.06 mg/ml. When the strain LZ-01 was pre-treated with 0.50 mM manganese, its ampicillin resistance level improved from 0.50 to 1.60 mg/ml; the chloramphenicol resistance level increased from 0.13 to 0.64 mg/ml (Fig. 4).

 When chromate and ampicillin were simultaneously added to cultures of strain LZ-01, the strain LZ-01 showed high co-tolerance level to chromate and ampicillin. The strain LZ-01 could resist to 6 mM chromate, and 0.75 mg/ml ampicillin in TSB medium. When the chromate concentration increased to 10 mM, strain LZ-01 still resist to 0.20 mg/ml ampicillin. When the ampicillin concentration increased to 1.25 mg/ml, strain LZ-01 still resist to 1.60 mM chromate (Table 4).

**Table 4:** Co-tolerance of different strains to Cr(VI) and ampicillin in TSB medium

Amp mg/ml		Cr(VI) mM							
		0.8	1.6	3.2	4	4.8	6	8	10
LZ-01	0.1	+	+	+	+	+	+	+	+
	0.2	+	+	+	+	+	+	+	+
	0.4	+	+	+	+	+	+	–	–
	0.5	+	+	+	+	+	+	–	–
	0.6	+	+	+	+	+	+	–	–
	0.75	+	+	+	+	+	+	–	–
	1.0	+	+	+	–	–	–	–	–
	1.25	+	+	–	–	–	–	–	–
*ΔemrB*	0.1	+	+	+	+	+	+	–	–
	0.2	+	–	–	–	–	–	–	–
	0.4	–	–	–	–	–	–	–	–
*ΔemrA*	0.1	+	+	–	–	–	–	–	–
	0.2	+	–	–	–	–	–	–	–
	0.4	–	–	–	–	–	–	–	–
CB	0.2	+	+	+	+	+	+	+	–
	0.4	+	+	+	+	+	–	–	–
	0.5	+	+	+	+	–	–	–	–
	0.6	+	+	+	+	–	–	–	–
	0.75	+	+	–	–	–	–	–	–
	1.0	+	–	–	–	–	–	–	–
	1.25	+	–	–	–	–	–	–	–
CA	0.2	+	+	+	+	+	+	–	–
	0.4	+	+	+	+	+	–	–	–
	0.5	+	+	+	+	–	–	–	–
	0.6	+	+	+	–	–	–	–	–
	0.75	+	+	–	–	–	–	–	–
	1.0	+	–	–	–	–	–	–	–
	1.25	+	–	–	–	–	–	–	–

**Fig. 5:**
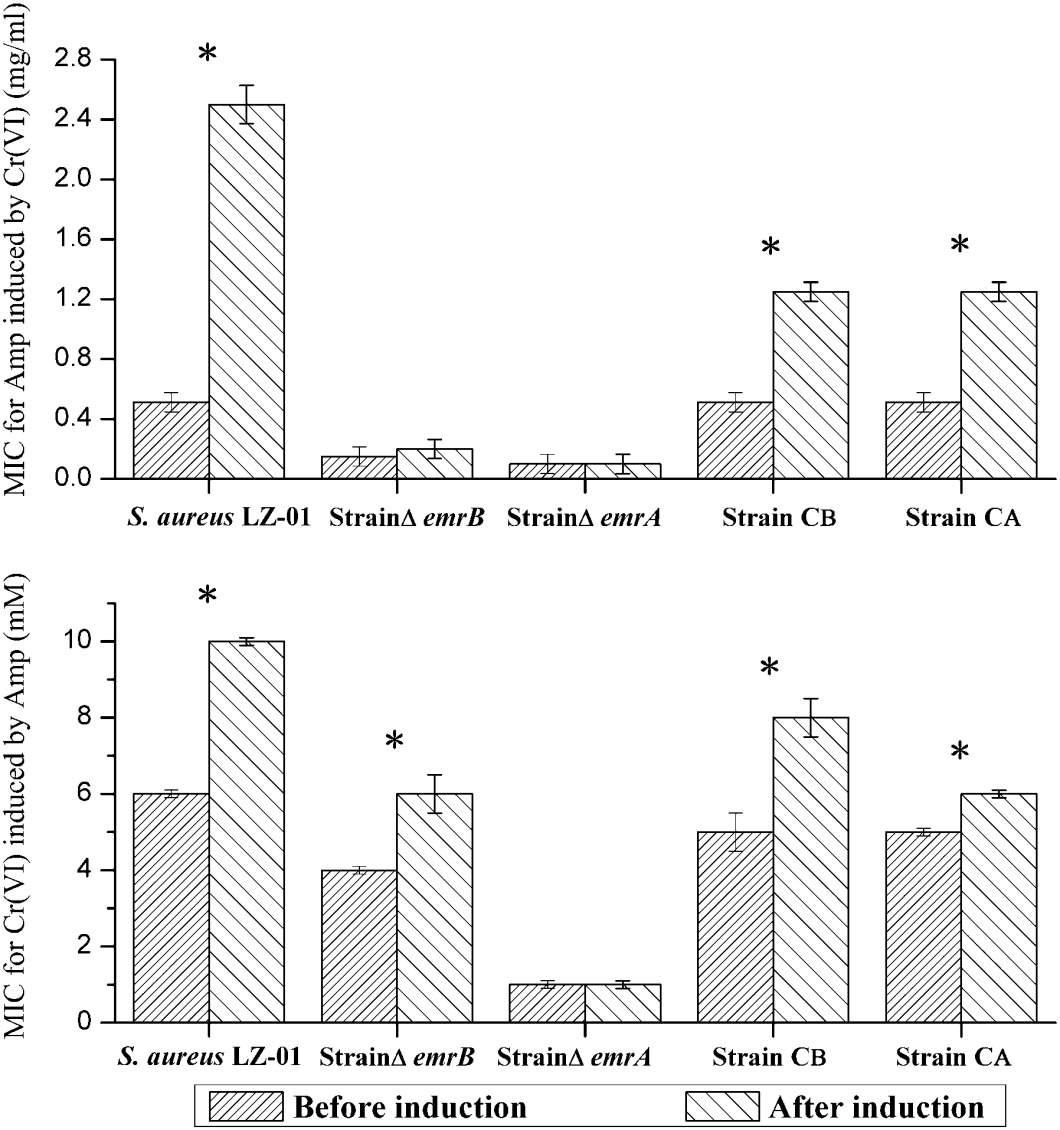
Induction effect of ampicillin and Cr(VI) on different strains. The induced level of Amp was 0.15 mg/ml for strain LZ-01, strain CA and CB; for mutant stain was 0.05 mg/ml. The induced level of Cr(VI) was 0.50 mM for all strains. *Asterisk* means significant difference between two groups (*T* tests, p < 0.05)

When the strain Δ*emrB* was induced by 0.50 mM chromate, its ampicillin resistance level could reach up to 0.20 mg/ml, with a 33 % increases; when the strain Δ*emrB* was induced by 0.05 mg/ml ampicillin, its chromate tolerance level could reach up to 6 mM, with a 33 % increases. The induction effect on strain Δ*emrA* was not as apparent as strain Δ*emrB*, whatever 0.50 mM chromate or 0.10 mg/ml ampicillin was used. The induction effect was more distinct on strain LZ-01, its induced ampicillin resistance level increased 5 times (from 0.51 to 2.50 mg/ml), and its induced chromate tolerance level increased 1.67 times (from 6 to 10 mM) (Fig. 5). The results of cross-tolerance tests of strain Δ*emrA* and Δ*emrB* to chromate and ampicillin were similar, only the ampicillin resistance level increased slightly, in comparison to their single resistance to chromate or ampicillin. The co-tolerance test of strain LZ-01 resulted in a remarkable difference, but this result was in accord with the induction findings (Table 4). The complemented strains CB and CA also showed higher co-tolerance level than mutants, but the co-tolerance level was still inferior to the wild strain LZ-01. These results revealed that co-induction phenomenon was existed in co-resistance which mediated by *emrAB* operon in *Staphylococcus aureus* LZ-01.

## Discussion

In this study, we proved the co-resistance of metals and antibiotics in *S. aureus* strain LZ-01. The RNA-seq and qRT-PCR methods were employed to find crucial genes that were up-regulated under chromate/ampicilllin stresses; the homologous recombination method was used for gene knockout. Our results indicated that a novel *emrAB* operon confers co-resistance of metal chromium and antibiotic ampicilllin in strain LZ-01. This *emrAB* operon encoded efflux pumps which belonged to the multidrug resistance (MDR) family, and was inducible by metals (chromate and manganese) and antibiotics (ampicillin and chloramphenicol).

The gene *emrB* of *Staphylococcus aureus* LZ-01 is 1948 bp in length, and EmrB protein belongs to a family of Major Facilitator Superfamily 1 (MFS_1) with membrane translocases and 14 transmembrane domains, in which many multidrug-resistant (MDR) proteins of Gram-positive bacteria (e.g., QacA, Rouch et al. 1990) and efflux pumps (e.g., TetA, Yamaguchi et al. 1990) are included. The EmrB protein of *Staphylococcus aureus* LZ-01 has another functional region that belongs to the EpsG family which involved in the production of exopolysaccharide (EPS) component of extracellular matrix during biofilm formation and maintenance (Barbe et al. 2009) (Additional file 1: Figure S2). EmrA is a periplasmic protein which often exists in gram-negative bacteria (Furukawa et al. 1993), and acts as a drug-binding protein that transfers drugs through cell membrane. A limited homology was found to members of HlyD family, including a component of the *E.coli* hemolysin efflux pump (Felmlee et al. 1985) (Additional file 1: Figure S3). This is the first report that EmrAB pumps involved in ampicillin and chromate co-resistance in *Staphylococcus aureus*.

The co-resistance of antibiotic and metal based on MDR pumps was widely found in various strains, and most pumps were capable to extrude toxic heavy metals and antibiotics out of cells, such as DsbA-DsbB system in *Burkholderia cepacia* and MDR efflux pumps in *Serratia marcescens* (Perron et al. 2004). But the resistance level coffered by MDR pumps was different, the chromate and/or ampicillin resistance level was quite small comparing with this study. *Salmonella abortus equi* was found to have high level resistance to arsenic (100 mg/ml), chromium (50 mg/ml), cadmium (100 mg/ml), mercury (5 mg/ml) and ampicillin (10 µg) (Ghosh et al. 2000). *Pseudomonas aeruginosa* EW32 isolated from southeastern Brazil showed co-resistance to tetracycline and copper, and antibiotic resistance could be induced by heavy metals in the environment (Martins et al. 2014). There are some MDR pumps found in *Staphylococcus aureus*, but none has the chromate or ampicillin resistance. The Smr pump from *S. aureus* extrudes small structure cations such as ethidium bromide and tetraphenylphosphonium (Paulsen et al. 1993), the Acr pump extrudes sodium dodecyl sulfate, acriflavine, novobiocin and rifampin (Poole et al. 1993), the QacA pump extrudes quaternary ammonium compounds (Rouch et al. 1990). The EmrAB pumps in *S. aureus* LZ-01 could resist to both ampicillin (0.51 mg/ml) and chromate (6 mM), and the resistance could be highly induced by each other.

Concerning toxic metal resistance, the most studied toxic metal ions were bivalent cations including Ag^+^, Cd^2+^, Co^2+^, Cu^2+^ Hg^2+^, Ni^2+^, Pb^2+^, Sb^3+^, Ti^+^ and Zn^2+^, few reports described the efflux of anions including AsO_2_^−^, AsO_4_^3−^, CrO_4_^2−^ and TeO_3_^2−^ (Silver 1996). Resistance to chromium has been observed in several microorganisms, the resistance often resulted from reduced uptake of CrO_4_^2−^, but it was hard to determine whether there was chromate efflux or a direct block on uptake (Silver and Phung 1996). The chromium resistance was usually conferred by *chrBACF* operon from the transposable element or chromosome in many studies. The chromium efflux pump *chrA* conferred as high as 50 mM of resistance level against chromium. The *chrBACF* operon was strongly induced by chromate or dichromate, and this operon also resist to superoxide due to the superoxide dismutase activity of *chrC* (Branco et al. 2008; Morais et al. 2011). The chromium efflux related pumps were encoded by *emrAB* in *Staphylococcus aureus* LZ-01, and this pump also extrudes manganese. The chromate reduction process was not observed in this study. Compared with the 50 mM resistant level of *chrBACF* operon, the *emrAB* of *S. aureus* LZ-01 only has weak chromate resistant capacity of 6-10 mM.

The ampicillin resistance of gram-positive organisms conventionally relies to the beta- lactamase activity or the PBP analogues (Berger-Bächi 2002), but none of the typical structures was found on operon *emrAB* from *Staphylococcus aureus* LZ-01. However, the expression of four PBPs was up-regulated under ampicillin stress, especially PBP1 (encoded by *pbpA*) and PBP4 (encoded by *pbp4*) (Table 3). In the mutant strain Δ*emrA*, all of the four PBPs levels were down-regulated after ampicillin induction, while PBP1 (encoded by *pbpA*) and PBP4 (encoded by *pbp4*) were slightly up-regulated in strain Δ*emrB* (Table 5), these results were consistent with the results of induction tests in Fig. 5. Therefore, the PBPs levels seemed to be responsible for ampicillin resistance in *Staphylococcus aureus* LZ-01, the inactivation of *emrAB* operon had a negative influence on the expression of PBPs.

**Table 5:** The expression level change of four PBPs genes under ampicillin stress (0.05 mg/ml) in *S. aureus* LZ-01 determined by qPCR

Genes	LZ-01	*ΔemrB*	*ΔemrA*	CB	CA
*pbpA*	3.88 ± 0.22	1.13 ± 0.15	1.07 ± 0.11	2.51 ± 0.16	2.44 ± 0.08
*pbp2*	2.02 ± 0.13	0.76 ± 0.07	1.38 ± 0.10	1.35 ± 0.09	1.63 ± 0.13
*pbp3*	2.71 ± 0.17	0.85 ± 0.12	0.26 ± 0.05	1.55 ± 0.10	1.47 ± 0.09
*pbp4*	3.32 ± 0.23	1.33 ± 0.05	0.45 ± 0.07	2.23 ± 0.12	1.89 ± 0.11

The induction effect on *emrAB* operon of *Staphylococcus aureus* LZ-01 indicated the existence of a regulation system, which sensed the inducer- ampicillin and chromium, and then regulated the expression of *emrAB*, and other resistant genes may also involved. A well-studied model for co-resistance of antibiotics and metals is the BaeSR regulation system. The BaeS may sense the presence of indole, copper and zinc, and then activates the expression of BaeR, which could bind to the promoter region of several mutidrug efflux pumps components including *mdtA*, *tolC* and *acrD*. BaeR induced the expression of mutidrug efflux pumps, thus conferring the resistances to novobiocin, deoxycholate and β-lactams in *Salmonella* (Nishino et al. 2007). CzcR-CzcS was another well-studied two-component regulation system involved in heavy metal and antibiotic resistance in *Pseudomonas aeruginosa*, sublethal zinc concentrations induced resistance to zinc, cadmium, cobalt and imipenem. The CzcR-CzcS systerm can sense the metals zinc and cadmium, and then regulates the expression of heavy metal efflux pump CzcCBA, as well as the imipenem-resistant antibiotic resistant porin OprD (Perron et al. 2004). The *emrAB* operon was often regulated by *emrR*, a MarR family transcriptional repressors (Lomovskaya et al. 1995). The repression of *emrR* could be relieved by structurally unrelated compounds such as uncouplers of oxidative phosphorylation, salicylic acid and 2, 4-dinitrophenol (DNP) (Hinchliffe et al. 2014). However, the *emrR* was not found in the transcriptome of *S. aureus* strain LZ-01, a homologous *marR* gene-*SAV2265*, which also acts as a negative regulator, was found in the upstream of *emrAB* operon. MarR also bound to the promoter region and flanked both -35 region and -10 region, and served as a repressor (Lomovskaya et al. 1995). The *marR* was previously reported as a copper sensor that regulates the multiple antibiotic resistances in *Escherichia coli* (Hao et al. 2014). So we primarily tested whether the *marR* gene can regulate the transcription of *emrAB* in *S. aureus* LZ-01. The MarR protein was added to the cultures that harboring promoter from *emrAB*, but no obvious regulation effect was observed (Additional file 1: Figure S4). For chromate resistance in *S. aureus* LZ-01, the *emrAB* pumps could extrude redundant chromate out of cell; but for ampicillin resistance, the *emrAB* pumps may mediate a regulation function that regulate the expression of *pbp* gene clusters. Besides, the *emrAB* pumps also contribute to the cell growth and proliferation, while the deletion of *emrA/B* have reduced cell density and delayed growth rate. Thus the growth defects induced by deletion weakened their resistance. As previously described, the primary mission for chromosomal MDR pumps is keep chemical equilibrium of cells, including but not limited to detoxification of metals or antibiotics. Recently, new functions of chromosomal MDR pumps were revealed, including biofilm maturation, virulence, quomm sensing and signal communication (Martinez et al. 2009).

## Conclusion

 Our findings here demonstrate that microorganisms not only have the specific pathway to resist to a poison substrate, but also have a broad-spectrum defense mechanism to resist various poison compounds. This point is especially valuable for new antimicrobial drug design, and new antimicrobial compounds should limit microbial activities other than synthesis and maintenance of cell walls. Meanwhile, the co-resistance of metals and antibiotics in *S. aureus* LZ-01 is confirmed in our study, and this co-resistance maybe based on the broad-spectrum defense mechanism, like biofilm formation and MDR efflux pumps. The contamination of environments will maintain and strengthen the effect of co-resistance based on transferable elements, even induce to the emergence of new resistance (Bos et al. 2015). The elimination of contaminants (metals, antibiotics, antimicrobials, etc.) and limited usage with caution are necessary for alleviating the co-resistance.

## Additional file

10.1186/s40064-016-3253-7 The background information for *emrAB* and its regulation test.
