# Supplementary material for: Multidrug resistance operon emrAB contributes for chromate and ampicillin co-resistance in a Staphylococcus strain isolated from refinery polluted river bank
Source: Springerplus. 2016 Sep 22;5(1):1648. doi: 10.1186/s40064-016-3253-7 (PMC5033799; doi:10.1186/s40064-016-3253-7)
Supplement: Supplementary file 1 — 10.1186/s40064-016-3253-7 The background information for emrAB and its regulation test. [file 40064_2016_3253_MOESM1_ESM.docx]

Supplementary Material for

Multidrug resistance operon *emrAB* contributes for chromate and ampicillin co-resistance in a *Staphylococcus* strain isolated from refinery polluted river bank

He Zhang^1^, Yantian Ma^1,2^, Pu Liu^1^, Xiangkai Li^1^

1. MOE Key Laboratory of Cell Activities and Stress Adaptations, School of Life Sciences, Lanzhou University, Lanzhou, Gansu, 730000, China
2. School of Life Sciences, Nanchang University, Nanchang, Jiangxi, 300031, China

Corresponding Author

Xiangkai Li

Key Laboratory of Cell Activities and Stress Adaptations, School of Life Sciences,

Lanzhou University, Tianshuinanlu #222, Lanzhou, Gansu, 730000, P.R. China

Tel: 86-931-8912561,

Fax: 86-931-8912560

E-mail: [xkli@lzu.edu.cn](mailto:xkli@lzu.edu.cn)

This file includes

Figures S1-S4

Tables S1

**Figure captions:**

Figure S1. The phylogenetic trees of *emrA* and *emrB* based on the amino acid sequences.

Figure S2. The conserved domains of *emrB* blasted in CDD of NCBI.

Figure S3. The conserved domains of *emrA* blasted in CDD of NCBI.

Figure S4. The expression level of GFP was affected by *MarR* protein, ampicillin and/or chromium. CK is the culture harboring pSB3K3-P*_emr_*GFP plasmid; P_20_ means 20 µl crude extract of *MarR* protein was added; Amp and Cr stand for the addition of ampicillin (4µg/ml) and chromium (0.2 mM).


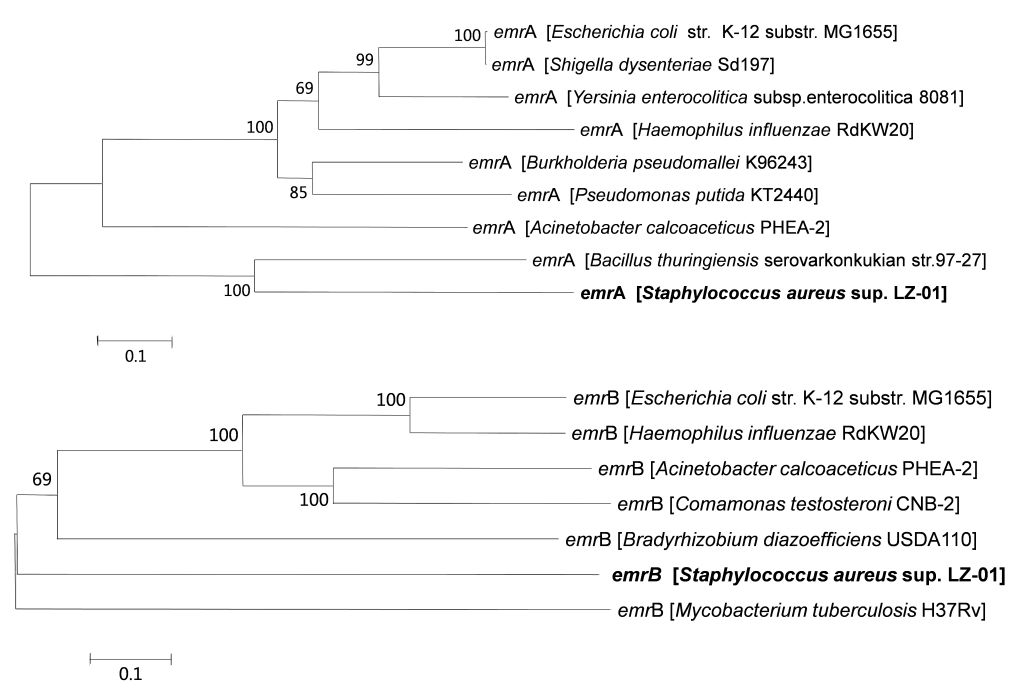


Figure S1


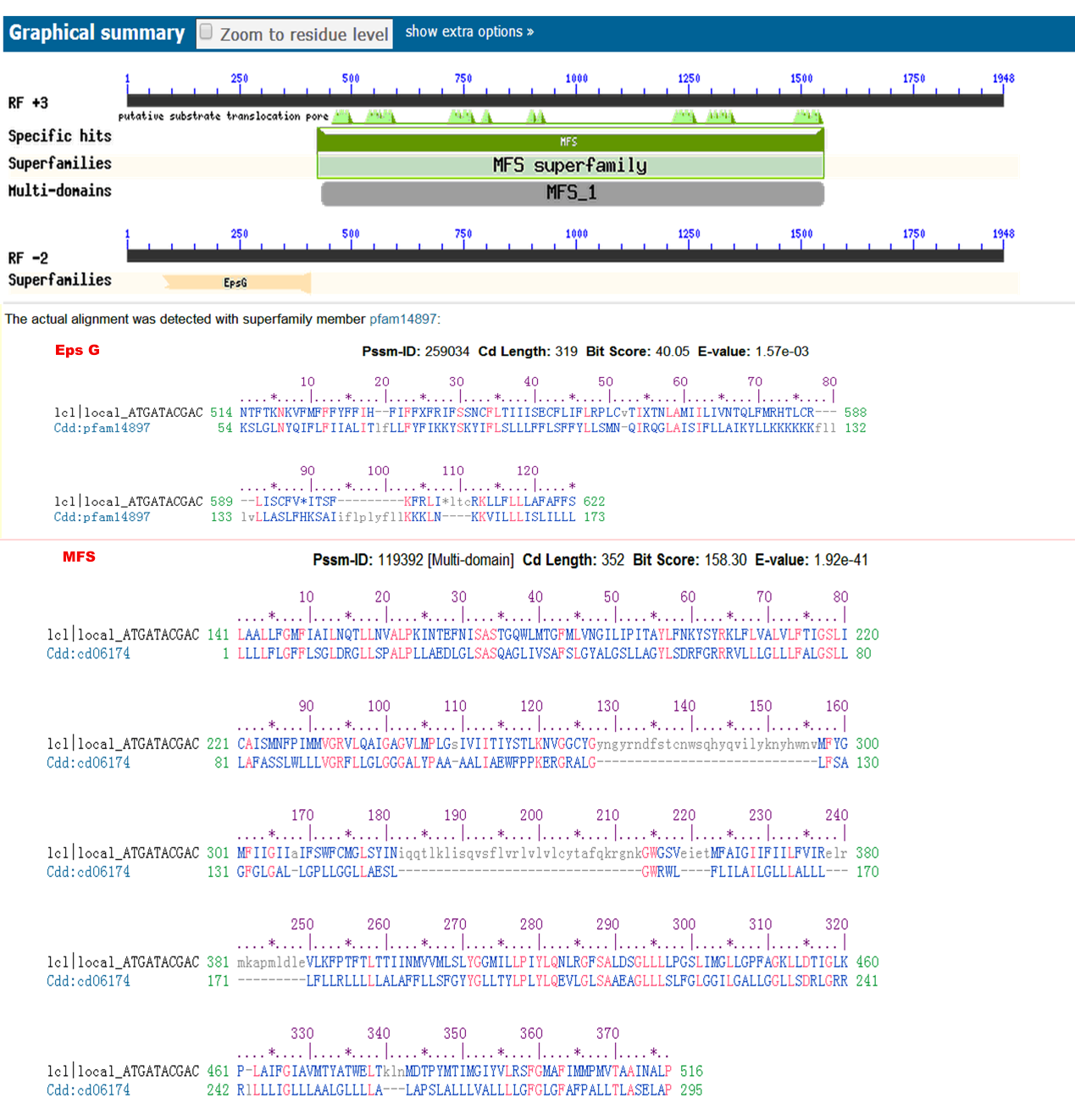


Figure S2


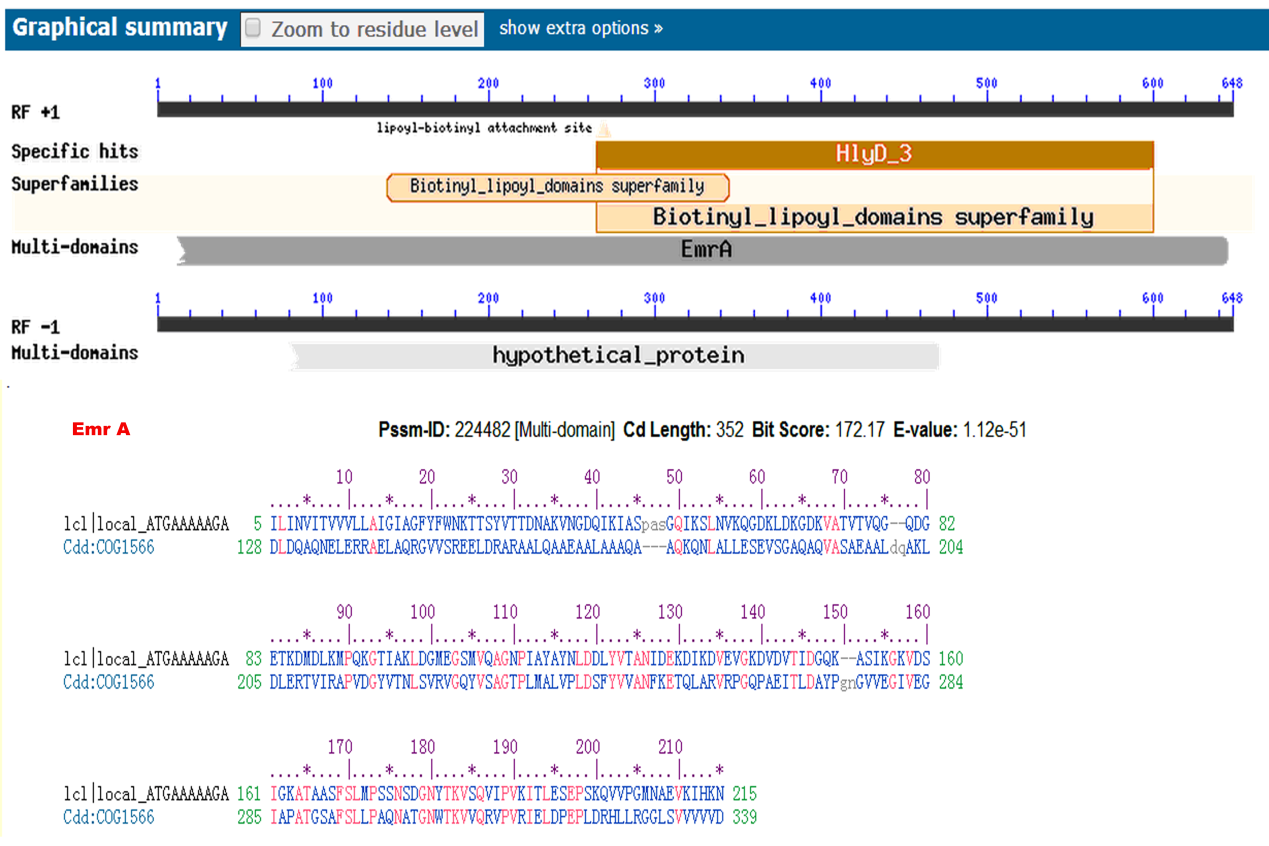


Figure S3


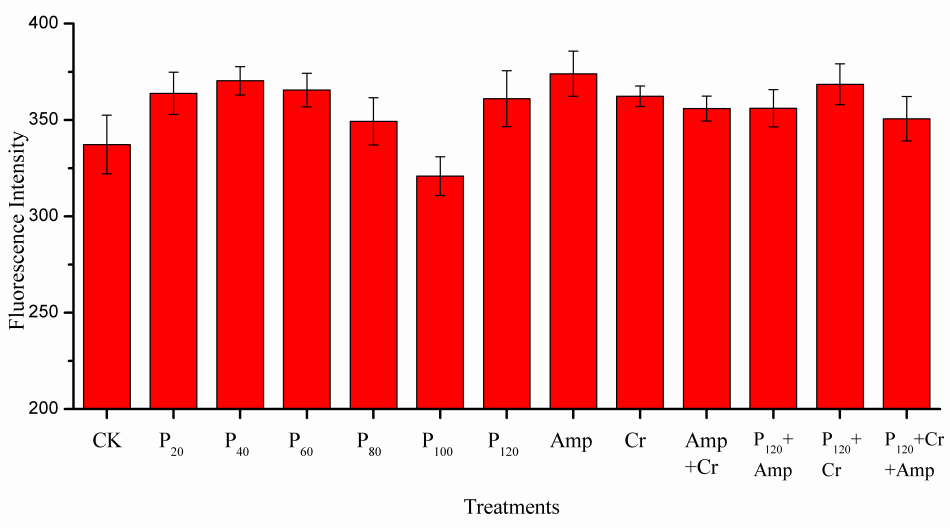


Figure S4

**The detailed procedure of Figure S4**

The sequence ahead of *emrAB* was identified and the promoter region was confirmed, which was 74bp long before the start codon. This promoter was named P_emr_, and was assembled into the reporter gene GFP by replacing the original promoter. Then the reconstructed P_emr_- GFP was inserted into the plasmid pSB3K3, the resulted vector pSB3K3- P_emr_GFP was transformed into *E. coli.* Top10 cells, the expression of GFP was confirmed by fluorescence microscope in positive clones.

The *marR* gene (SAV2265) was ligated into plasmid pET-28a on the *Nde*I and *Xho*I sites. The reconstructed plasmid was introduced into *E.coli.* DH5α and harvested, then transformed into *E.coli.* Rosseta (DE3) for the expression of *marR*. Chloramphenicol (30µg/ml) and kanamycin (50µg /ml) were added for screening. Before the extraction of *marR*, 0.5mM IPTG was added for induction and lasted 20 hours at 16 °C. The extracted *MarR* protein was verified by SDS-PAGE (17.2 KD).

Then the *MarR* protein was added into the cultures that harboring pSB3K3- P_emr_GFP, and the expression level of GFP was measured by fluorescence spectrophotometer. The effects of ampicillin (4µg /ml) and chromium (0.2 mM) were also concerned.

Table S1 The primers used for qPCR.

| Genes | Forward primer | Reverse primer |
| --- | --- | --- |
| 16S | CTCGTGTCGTGAGATGTTGG | TGTCACCGGCAGTCAACTTA |
| SAV0198 | ATGAATACGATAGAACGAAAGAA | GTTGTTGACCGCCTGATAGT |
| SAV0199 | ATTGAGGGACAGGGATTTGAA | ATTGAGGGACAGGGATTTGAA |
| SAV0203 | TGAATAATGTGAATGCTAAGTT | TACATCACCTTTTGTTGGCA |
| SAV0274 | TCTAGGCGTTACAGTGGTCTTT | CCTAAACGAATCATCGCAACT |
| SAV0275 | GCACAGGATTCACAATACAAACT | TGCGAGATGTCCAACGATAA |
| SAV0276 | TCAGCAAGTAAAGCGACAGC | CACCACCACCGTGATATTGT |
| SAV0277 | TTGGTGATAATGGTGCTGGAA | AATAACGCAATGTCTTGTGGC |
| SAV0351 | CAAGAGGGATGCGAATGAA | CCTCCACCTTGAGCGACA |
| SAV0661 | CATAATGTTACCACTAACGGTTCA | CAGATGTTCGTTGTCTTTGTCC |
| SAV1035 | AAGTTCATACGCTAAGTGGAGG | GTTTTAGGATCTAACGCACCC |
| SAV1318 | TGGTGCTGGAAAGTCAACG | TTTGGAACATTATACTGCGATT |
| SAV1837 | TGGAAAACGCCCTGTAATT | CCTTCCATAGGTGTAAGCAATC |
| SAV1866 | GGTCGTCCAACAGCAACAGA | ATAATTTAACACCTCGTTCACCT |
| SAV2166 | ATTGCTTTAGTTGCCTTTGTTT | GCGTATAGCATTAACCCGAATC |
| SAV2168 | ATCTTGGGGCATCAATAGGT | GCTAATTACAGCAACAGCACTTA |
| SAV2169 | GTTGGCAGTGTTTGGGGTAT | GCAATGATCGCAATCGGTA |
| SAV2261 | CTATTGTATTTGGAGGACCGAG | CATGATTCAACACTGCTTGCT |
| SAV2262 | TCTGAACATTATGGCTTATGGG | TGGTACAATGGCTGATTCAATAT |
| SAV2265 | CGCTAAACATCAGCCCACTA | AACTTTTGTCGTTTATCCTCTAAA |
| SAV2266 | ATTTTCACCAAAAGTTTTACAATC | AATGAATCGGAAGCGTCG |
| SAV2352(*emrB*) | ACGACACGTCAATCAAATCAA | CAACACGCAACTCTTCATGC |
| SAV2353(*emrA*) | CAAGATGGCGAAACGAAAG | GGTTACCAGCTTGCACCATT |
| SAV2355 | AAACAACGACGTCTAACGCAC | CGCCCAGTTGAATCTGAAAT |
| SAV2419 | TAAATGGGGAGTGAAAGCAA | TGATTATCTGGGACGAAATAGTC |
| SAV2420 | GCTTACTTGCCCCTCTTGC | TCGCTTCCTTTACCGATGT |
| SAV2421 | CTGCTAATGCTGAAGGTAAAATAA | CAGAATCTGCTGTGGCTGTT |
| SAV2428 | TTGTTTGGCAATCCAGGTG | TGTTTGATATCCCTCAGGTAATG |
| SAV2462 | ATTATTGCTCGTGTCGGTCC | TGCTACGAAATAACCTTCTAATCC |
| SAV2552 | GCAATAGGAACAGCAGGTAGT | ACCCCATCACTGCTAAGAAAT |
| SAV2623 | GTGAGTCTGGGTCAGGGAAG | GTTGACGATACAATGCTTTACTTT |
| SAV2701 | TTCGTTGCGATTATGGGG | AAGTTTATTAAGCTCTTGTCCGTT |
| SAV2702 | AACCCTTACATCTATGGCACC | ATTTGTTGTTGGCTTAGTTTAGTT |
| SAV1275 | GAAGGCGTATTTGTCTTAATCAG | TGAAACAATCAAGCGACCTC |
| SAV1504 | AACAGGGTCCAGTTGTAGCA | CATGTACCATCACATCAGCATC |
| SAV1545 | GCTGTTATTCTGATTGACCCTT | CATCGACTGCTCCGATATGAT |
| SAV2441 | CATCAACTGAAGATTTGGAACA | TAAACCAGCCCGAAGCATA |
| *pbpA* | TTGGATGGAGTAATGAGTTGC | CGCTGATTGCGCCTGTA |
| *pbp2* | TATGAAATGGGCAACAAACC | ACAGTACCGTGACTCTTCGTATC |
| *pbp3* | CGGAAAGCAGATTAATAAGAGTG | CAGAAGATCCAACCGCAAA |
| *pbp4* | AACCATACGATTACTACCAAACG | GCTTCTCGCCACCAAGG |
